# Colonization of Different Grapevine Tissues by *Plasmopara viticola*—A Histological Study

**DOI:** 10.3389/fpls.2019.00951

**Published:** 2019-07-24

**Authors:** Sarah Fröbel, Eva Zyprian

**Affiliations:** Julius Kühn-Institut, Institute for Grapevine Breeding Geilweilerhof, Siebeldingen, Germany

**Keywords:** downy mildew, host-pathogen-interaction, infection structures, oomycetes, *Vitis vinifera*

## Abstract

*Plasmopara viticola*, the downy mildew pathogen, is one of the most important pathogens in European viticulture. This oomycete infects grapevine leaves via zoospores that encyst at stomata. A primary germ tube then enters the substomatal cavity and develops a tubular network of hyphae that proliferate intercellularly and parasitize the leaf mesophyll cells by haustoria. Leaf infections have thus been the primary object of multiple studies concerning the physiology of the pathogen and defense reactions of grapevines. Besides leaves, this oomycete pathogen is able to spread throughout the plant tissue. As shown here by microscopy, it colonizes leaf petioles, shoots, berries and seeds. Evidence is provided showing that this process is facilitated by formation of special fan-shaped hyphae that seem to be necessary to overcome physical barriers in plant tissues. Physical obstacles are mainly constituted by vascular tissue in leaf veins, leaf petioles and shoots. In grapevine shoots, the mycelium seems to extend along the cambial layer between xylem and phloem tissue. Infected young berries are completely colonized on the inside. Older infected “leather berries” show glossy appositions of the fan-shaped hyphae at the inner side of the berry skin. The seeds from that stage of infestation are devoid of endosperm and embryo and biologically dysfunctional. Furthermore, a classification system for *P. viticola* infection based on the degree of infections in petioles and shoot tips is presented. This study contributes to a better understanding of downy mildew pathogenesis in grapevine, a prerequisite for efficient control measures.

## 1. Introduction

*Plasmopara viticola* [(Berk. & Curt.) Berl. & de Toni], belonging to the order of *Peronosporales*, is an obligate biotrophic oomycete pathogen of grapevine and causes downy mildew (DM) (for review see Gessler et al., 2011; Fawke et al., 2015; Kassemeyer et al., 2015). It is one of the most dangerous pathogens in viticulture. Since its accidental introduction from North America in the late 1870s, it causes considerable damage in European vineyards (Kassemeyer et al., 2015). It strongly contributes to the necessity to apply large amounts of fungicides to protect the plants and their fruits. Heavy chemical application is in conflict with the requirement for sustainable and environment-friendly agriculture. Current grapevine breeding therefore introduces genetic loci mediating *P. viticola* resistance from several *Vitis* wild species of North American or Asian origin into new cultivars of *Vitis vinifera* (Töpfer et al., 2011). Around 25 different loci encoding resistance factors to *P. viticola* have been described (http://www.vivc.de/ data on breeding and genetics; direct link https://bit.ly/2L1k6KX). However, individual resistance loci may be overcome by adaptation of the pathogen (Kast, 2000; Peressotti et al., 2010). This problem results in the continuous search for new resistance loci to be “stacked” into new cultivars by crossbreeding (Eibach and Töpfer, 2015) for durable resistance to DM.

*Plasmopara viticola* infects its host by biflagellate zoospores released from asexually produced sporangia. Splashing water distributes the sporangia to the underside of grapevine leaves and other parts of the plant. In the presence of moisture, the zoospores swim in a targeted manner to stomata, where they encyst at the rim and develop a germ tube that enters the substomatal cavity. A substomatal vesicle is formed which starts proliferation of the pathogen with a diploid, non-segmented tubular mycelium. It grows intercellularly and retrieves nutrients by parasitizing the host cells via haustoria. Haustoria develop beneath appressoria and arise from penetration pegs. After ~5 days under optimal conditions at around 25°C, the mycelium produces sporangiophores that grow out of stomata and discharge new sporangia at their tips, ready to spread the pathogen. This asexual propagation cycle is responsible for DM epidemics arising each season in warm and humid weather conditions. It is complemented by sexual propagation in this heterothallic organism through meiosis and oogametangiogamy in late summer. Rigid oospores are formed that survive winter conditions in the soil or decomposing leaves. The oospores develop primary sporangia in spring at moist conditions and rising temperatures (Gessler et al., 2011; Kassemeyer et al., 2015). The primary sporangia release zoospores to re-start the epidemic by infection with genetically recombined strains. Primary oospores may also contribute considerably to the epidemics that develops during summer (Rossi et al., 2013). Research on resistance typically involves phenotyping assays using leaf explants (leaf discs) that are artificially inoculated with DM isolates from field collection or propagation in greenhouse conditions on susceptible grapevines (e.g., Brown et al., 1999; Deglène-Benbrahim et al., 2010). The severity of the infection can be scored a few days after inoculation. However, these experiments do not reflect infestation strategies of the pathogen conquering larger areas of the leaf or other plant organs. They are unable to reveal possible defense responses operating outside of the dissected leaf tissue. Knowledge on the progress of infection in other parts of the plants is scarce. Here we present a histological study about the infestation of not only leaves but also other organs of grapevines, such as shoot tips, petioles, berries and seeds. This histological investigation aims to improve our understanding of the pathogenesis of DM. It provides the basis to study plant reactions operating in resistant grapevine genotypes beyond the leaf tissue. This is important, since in most cases of DM resistance identified in North American or Asian *Vitis* species and applied in resistance breeding, the pathogen is still able to enter the mesophyll through stomata and to establish the first infection structures (Unger et al., 2007; Diez-Navajas et al., 2008; Jürges et al., 2009). Resistance relies on following diminished propagation mediated by some—not yet fully understood—“post-penetration” mechanisms.

## 2. Materials and Methods

### 2.1. Plants and Pathogens

Initially, plantlets of two susceptible grapevine cultivars (“Riesling” and “Müller-Thurgau”) were obtained by cutting woody 1 year old shoots stored in the cold for 1–2 months into sections of ten to 15 cm with two buds, one at each end. The cuttings were rooted in Jiffy pots (Jiffy7®, Jiffy products International BV, Zwijndrecht, Netherlands) and maintained in 10 replicates per genotype after planting in soil (Hawita Fruhsdorfer Poinsettienerde, Gärtnereinkauf Münchingen GmbH, Korntal-Münchingen, Germany) in 12 cm pots. At the stage of 4–6 unfolded leaves, the plants were transferred to the climate chamber for 1 week before experimental inoculation. The climate chamber was operated at 80% relative humidity with 16 h day light (65 klux) at 23°C and 8 h night at 16°C. The plants were watered twice a week without fertilization over the complete test period of 3 months (in the greenhouse and afterwards in the climate chambers). They were experimentally inoculated (see below) and leaves were collected at 6 days post-inoculation (dpi).

The microscopic evaluation was extended to plants grown outside in the field under natural infection pressure to confirm the phenomena seen in experimentally inoculated cultivars from the greenhouse. The range of cultivars was broadened. Besides “Riesling” and “Müller-Thurgau” the susceptible cultivars “Pinot blanc” and “Pinot noir” and one DM resistant grapevine cultivar [“Solaris”—carrier of resistance loci *Rpv10* and *Rpv3.3*, (Schwander et al., 2012)] maintained in the field plantation of the Geilweilerhof Institute (geographic position 49_21.7470 N, 8_04.6780 E) were investigated. These scion cultivars are grafted on rootstocks Kober 125 AA (*Vitis berlandieri* × *Vitis riparia*) in the cases of “Pinot noir” and “Pinot blanc” (*n* = 50 per cultivar), respectively SO4 (Selektion Oppenheim 4; *Vitis berlandieri* × *Vitis riparia*) for the susceptible cultivars “Riesling,” “Müller-Thurgau” (*n* = 20 per cultivar) and the resistant cultivar “Solaris.”

The plants are trained in a vertical shoot positioned trellis, a traditional system in Germany and in the region (Palatinate). The distance between the rows is two meters; the distance between the individual vines extends over one meter. For experimental purposes, the plantation was left unsprayed without any plant protection since 4 years. In the Palatinate region, due to warm and moist weather conditions, there is a high natural infection pressure of DM arising every season.

Leaves (*n* = 100), petioles (*n* = 104), shoot tips (*n* = 51), berries and seeds (*n* more then 100) were studied in naturally infected field-grown plants. The vegetative organs (leaves, petioles, shoot tips) were evaluated by microscopy during the period from April to August 2018. The first examination of infected berries and seeds was before ripening (June 11, 2018), when the individual berries were still firm and the stomata still open (BBCH 71, Lorenz et al., 1994). DM-infected berries were also collected later at véraison stage (BBCH 81, Lorenz et al., 1994) in August 2018. At that late stage, they had turned into destroyed “leather berries,” characteristic of earlier *P. viticola* infection. The resistant field-grown cultivar “Solaris,” did not show any symptoms of infection (no oil spot, no sporangiophores at the lower site of the leaves, no infected berries) with *P. viticola*.

Leaves (*n* = 100) with typical symptoms of DM of the susceptible cultivars were chosen to study the corresponding petioles. A selection of leaves (*n* = 20) from “Solaris” (showing no DM symptoms) were collected for comparison. The petioles were detached from the (infected) leaves. They were cut twice longitudinally or transversally with a razor blade. For assessment of the colonization of shoot tips, shoots were selected in which at least one leaf was obviously infected with *P. viticola* from the susceptible cultivars. For “Solaris” randomly selected shoot tips of four different individual plants were cut-off. The leaves were removed from the growing tip and the lower part of the shoot tip (at 20–30 cm from the top) was cut twice longitudinally or transversally. For evaluation of berries (*n* = 100, taken from different grape clusters), infected and uninfected field plant berries were collected and cut into fine slices for microscopy. “Solaris” did not show any symptoms of infection on the berries throughout the year, thus uninfected berries were collected and prepared for comparative microscopy. For the investigation of seeds, infected resp. uninfected (without any macroscopic symptoms of infection) berries of the susceptible cultivars (“Riesling,” “Pinot blanc,” and “Pinot noir”) and of the resistant cultivar “Solaris” were sampled during the two aforementioned stages. The seeds (more than 100) were separated from fruit flesh and were on one hand cut longitudinally and on the other hand cut transversally, using a razor blade.

### 2.2. Experimental Inoculation

Whole plants (“Riesling” and “Müller-Thurgau”) in the greenhouse were inoculated by spraying a suspension of freshly prepared sporangial solutions. Inoculum was obtained from leaves of “Pinot blanc” field plants with typical oil spot symptoms and a whitish layer of sporangia on the lower leaf. Sporangia were collected by vacuum sucking over sporangial layer on the leaf with a water jet pump connected to a pipette tip. The collected sporangia were suspended in 30 ml water and the suspension was adjusted to 30,000 sporangia per ml before incubation at room temperature for 1 h to release the zoospores. The plants were spray-inoculated on the lower leaf side until run-off and incubated in the dark overnight.

### 2.3. Histological Staining and Microscopy

Samples for microscopy were bleached overnight at 65°C in 1N KOH. Petioles and excised parts of shoot tips were incubated in 10 ml of KOH, while larger leaf sections (2 cm^2^) were kept in 30 ml of KOH. The samples were further washed three times in distilled water and stained with 0.05% (w/v) aniline blue in 0.067 M K_2_HPO_4_ (pH 9) (Hood and Shew, 1996) for at least 10 min. Microscopic evaluation of the leaves from the greenhouse plants (“Riesling” and “Müller-Thurgau”) followed at 6 days post-inoculation. For the natural infections from the field plants (“Riesling,” “Müller-Thurgau,” “Pinot blanc,” and “Pinot noir”) microscopy was done by collection of the different parts of these plants, KOH treatment and staining as above. Microscopy used an epifluorescence microscope Leica DM4000B-M (excitation at 395–440 nm, emission filter 470 nm). Stereoscopic views were taken with stereomicroscope Leica M205FA.

### 2.4. DNA Extraction

DNA was extracted from every organ sampled from the infected plant material and investigated by microscopy using the NucleoSpin®Plant kit (Macherey-Nagel, Düren, Germany). DNA was extracted according to the protocol supplied by the manufacturer.

DNA from *P. viticola* sporangia was prepared based on a slight modification of the protocol of Pintye et al. (2012). Frozen spores were crushed with a sterile conical grinder in 150 μ*l* of extraction buffer. The resulting suspension was kept at −20°C for 10 min and was then shifted to 70°C for further 10 min. After cooling down on ice for 5 min, it was centrifuged for 10 min at 12,000 g and room temperature. All further extraction steps followed the protocol. Final elution was in 50 μ*l* of TE buffer (10 mM Tris-Cl, 1 mM EDTA, pH 8.0). All DNA samples were stored frozen at −20°C.

### 2.5. Analysis of rDNA “Internal Transcribed Sequence” (ITS) for Pathogen Identification

A 20 μ*l* PCR mix was prepared under sterile conditions. ITS-flanking primers ITS6 and ITS7 described for *Peronosporaceae* (Cooke et al., 2000) were employed. Their alignment to the *P. viticola* sequence (Yin et al., 2017) was checked. The primers ITS6 and ITS7 match perfectly and amplify a 314 bp product from the “Internal Transcribed Spacer” (ITS) of *P. viticola* rDNA. PCR was performed using Phusion™ polymerase (Thermo Fisher Scientific, Waltham, MA USA) at an annealing temperature of 61.5°C with 35 cycles for amplification and an elongation time of 15 s. 1 μ*l* of undiluted DNA extraction provided the template. The positive control (DNA extracted from sporangia) was diluted 1:10 and 1 μ*l* of this dilution was used as template. The PCR products were characterized by their size (as estimated on 3% agarose gels run in 1 × TAE [40 mM Tris-acetate, 1 mM EDTA, pH 7.9, stained by Serva DNA Stain Clear G (Serva GmbH, Heidelberg, Germany)]. The fragments were cut out from the gel, purified with a NucleoSpin® Gel and PCR Clean-up Kit (Macherey-Nagel, Düren, Germany), and their identity confirmed by sequencing (Eurofins Genomics, Ebersberg, Germany).

## 3. Results

### 3.1. Infestation of Leaves

**Figure 1:**
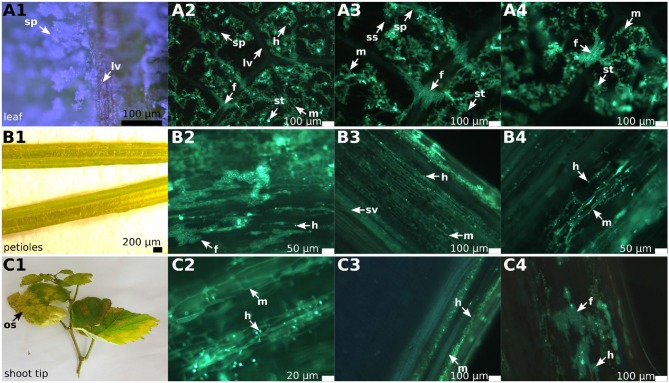
Overview of DM infected leaves, petioles and shoot tips in grapevines. The picture shows an example of infected “Pinot blanc” leaves, petioles and shoot tips. The left row of this panel shows a binoscopic overview (1) of the indicated plant organ, followed by three microscopic pictures (2–4) to the right. The samples were treated with KOH, stained with aniline blue and analyzed by epifluorescence microscopy. **(A1–A4)** Leaf segments of the susceptible grapevine infected with *P. viticola*. The binoscopic image **(A1)** shows the typical growth of the pathogen on the lower side of the leaf after 6 days of pathogen development. The microscopic images **(A2–A4)** show the clear separation of the intercostal fields by the leaf veins and the infiltrating *P. viticola* mycelium. On several leaf veins, the crossing by fan-shaped hyphae and the continuous spreading of the hyphae to the adjacent intercostal field is visible. **(B1–B4)** Not only the leaf veins can be overcome, but *P. viticola* also appears to spread into the petioles. The mycelium **(B2–B4)** shows a rather straight longitudinally extended growth along the conductive tissue and forms a multitude of haustoria. Increased numbers of fan-shaped hyphae are evident. **(C1–C4)** The longitudinal section through the shoots tips also shows straight longitudinal growth of mycelium **(C2–C4)** with enhanced formation of haustoria and the development of fan-shaped hyphae. sp, sporangiophores; m, mycelium; lv, leaf veins; f, fan-shaped hyphae; h, haustoria; sv, spiral vessel; ss, sporangia; st, stomata; os, oil spot.

Early in the year (April 10, 2018), first histological analyses were performed on the two susceptible genotypes “Riesling” and “Müller-Thurgau” cultivated under optimal and constant conditions in climate chambers using experimentally inoculated plants. Their leaves collected 6 days after inoculation showed the typical symptoms of DM infection (oil spots on the upper side and whitish lawns of sporangia on the lower side of the leaf). Microscopy indicated the formation of the well-known tubular hyphae with haustoria in the leaf mesophyll and the formation of sporangiophores. In addition to the regular mycelium, special “fan-shaped” hyphae traversing some leaf veins within the samples were observed. This structural change of hyphae from tubular to fan-shaped was specific for the crossing of leaf veins. After passing the veins, the adjacent intercostal fields were colonized by the pathogen and the hyphae re-took their typical tubular appearance. The two different susceptible grapevine cultivars investigated after experimental inoculation showed the same pattern of formation of these vein-traversing special hyphal structures. With the beginning of the first natural infections in May 1, 2018, the leaves (*n* = 50) of field-grown plants of the two susceptible grapevine genotypes “Riesling” and “Müller-Thurgau” were studied in addition to “Pinot noir” and “Pinot blanc.” The natural infections occurred in every susceptible cultivar in the vineyard. In contrast, the resistant cultivar “Solaris” did not show any natural infection on all of its plant organs throughout the season (Figure S1). The field plants are grown in an experimental plot of the Institute and did not receive any plant protection. The leaves of the susceptible cultivars showed the typical signs of DM infection. Oil spots, tubular hyphae and the formation of sporangiophores were evident (Figures 1A1,C1). Again, fan-shaped hyphae (Figures 1A2–A4) and the resuming of regular tubular growth after crossing the leaf veins were observable (Figures 1A2–A4). The formation of fan-shaped hyphae and the spread of the pathogen from one intercostal field into adjacent intercostal segments on leaves occurred in all the samples of the susceptible cultivars investigated. The special fan-shaped hyphae in the susceptible cultivars showed no haustoria (Figures 1A2–A4) and no development of sporangiophores in the leaves, while haustoria and sporangiophores were frequent in the intercostal fields infected by the regular tubular mycelium. As “Solaris” showed no symptoms of infection, some leaves were randomly collected for microscopic analysis for comparison. No hyphal structures were visible.

### 3.2. Molecular Confirmation of *P. viticola* Identity

The identity of the fungal-like structures observed in microscopy as elements of *P. viticola* was first confirmed on molecular level in infected leaves of the grapevine cultivars maintained in the climate chambers. These plants were experimentally inoculated in spring. After 6 days, the inoculated leaves were removed and split into two halves: One-half of each leaf was stored frozen for DNA extraction and PCR amplification of the ITS region, while the other half was evaluated by microscopy. The PCR products matched perfectly to *P. viticola*-specific [*P. viticola* genome, GenBank ID MTPI00000000 (Yin et al., 2017)] ITS sequences in size and sequence (Figure S2).

**Figure 2:**
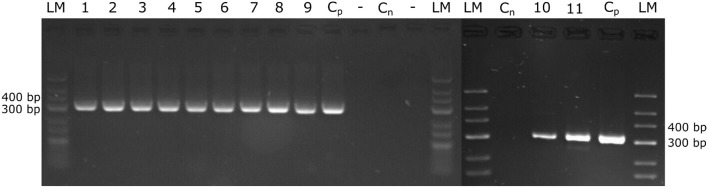
Amplification of the *P. viticola* ITS region. DNA was extracted from petioles and from shoot tips that were evaluated from the natural infected field plants by microscopy. The extracted DNA served to amplify the ITS-region of *P. viticola*, expecting a specific fragment size of 314 bp. The amplified PCR fragments were separated on 3% agarose-gels. The size standard applied is “GeneRuler Low Range DNA Ladder” (Thermo Scientific™). Cp, positive control; Cn, negative control; 1, 2, 3, “Pinot noir”; 4, 5, 6, “Müller Thurgau”; 7, 8, 9, “Pinot blanc”; 10, 11, “Riesling”.

In addition, excised parts of the microscopically examined petioles and shoot tips from infected field plants were stored frozen (−20°C) and used for DNA extraction later on, followed by PCR amplification of the ITS-region. All microscopically positive samples were also positive in the PCR assay, while the microscopically infection-negative samples were also negative in PCR. All amplified ITS-Fragments (Figure 2) from the various grapevine cultivars employed in this study were sequenced and found to be identical to *P. viticola* ITS-region.

### 3.3. The Infestation of Petioles and Shoot Tips

**Figure 3:**
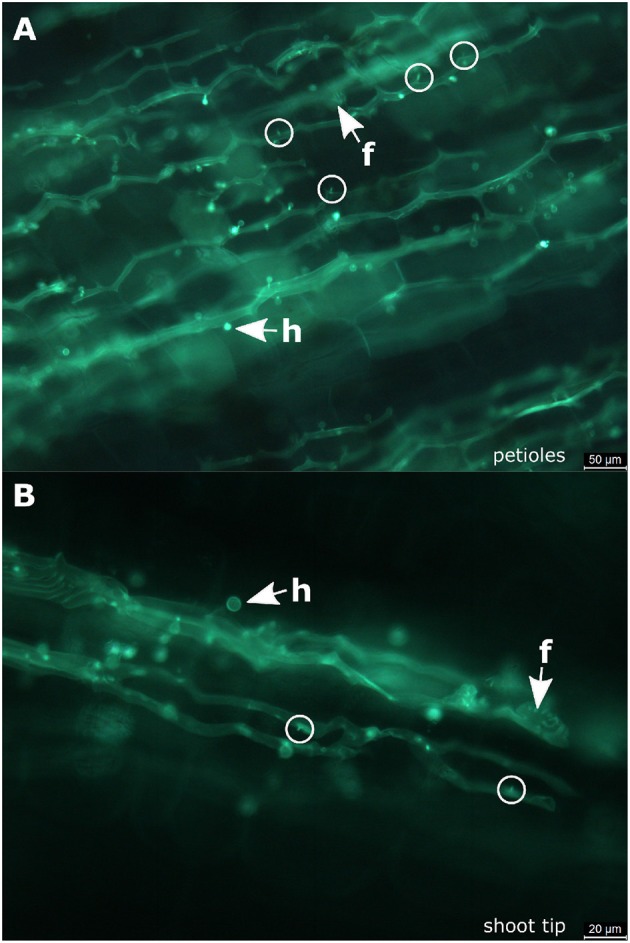
Infected petioles and shoot tips. **(A)** Microscopic image of an infected petiole from “Pinot blanc.” The hyphae follow the elongated structure of the organ and form numerous haustoria. At some positions, it is possible to observe the beginning of haustoria formation starting from small pegs (circle). **(B)** Microscopic picture of an infected shoot tip. Besides a longitudinal course of the regular hyphae, the fan-shaped hyphae are observed. The initial stages of haustoria formation are high-lighted by circles. f, fan-shaped hyphae; h, haustoria; circle, penetration peg.

**Figure 4:**
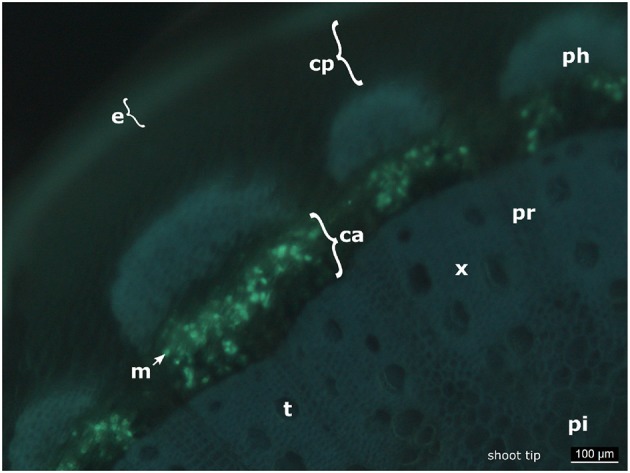
Cross section of an infected shoot tip. The picture shows a cross section of an infected shoot tip from “Pinot blanc” and the presence of the pathogen apparently in the cambium. e, epidermis; cp, cortical parenchyma; ph, phloem; m, mycelium; ca, cambium; x, xylem; pr, pith radiate; pi, pith; t, tracheae.

**Figure 5:**
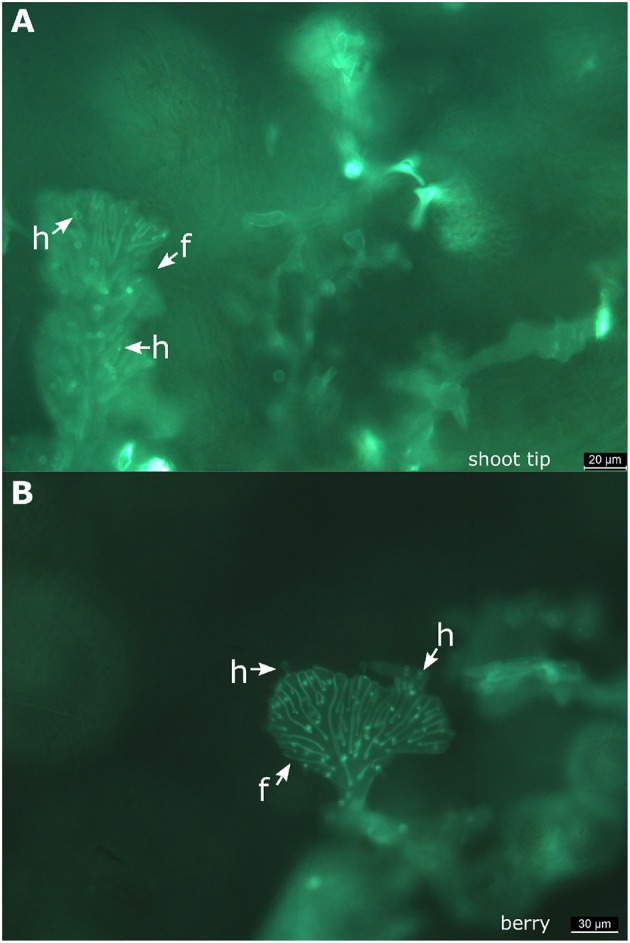
Fan-shaped hyphae forming haustoria. Microscopic views taken from a shoot tip **(A)** and from a berry **(B)** of “Müller-Thurgau.” It is evident that the fan-shaped hyphae of *P. viticola* produce haustoria in these tissues. f, fan-shaped hyphae; h, haustoria.

In the petioles and the shoot tips of the susceptible cultivars the development of mycelia with haustoria could be clearly recognized (Figures 1B1–B4,C1–C4, 3A,B). It follows the longitudinal extension of these plant tissues and apparently grows along the vascular elements. The formation of penetration pegs, which are the primordia of haustoria, is visible. The length of the penetration pegs is two μm. This structure enlarges and grows into young haustoria (Enkerli et al., 1997). In cross sections of the shoot tips, the mycelium appears to spread in the cambium layer between phloem and xylem (Figure 4). Formation of the special fan-shaped hyphae could be observed (besides the regular tubular hyphae) in all investigated tissues of the different grapevine genotypes. In the petioles (Figure 3A), the shoot tips (Figures 3B, 5A), the berries and the seeds (as shown below) these structures also formed distinct haustoria (Figures 5B, 8A2,A4,B2–B4), like the tubular hyphal structures. In contrast, in the passage of leaf veins, no formation of haustoria emerging from the fan-shaped hyphae could be detected (Figures 1A2–A4).

The resistant cultivar “Solaris” did not show any infection structures of *P. viticola* in the investigated petioles or shoot tips on every sampling date throughout the complete investigation period in the year 2018.

### 3.4. Classification of the Degree of Infestation

**Figure 6:**
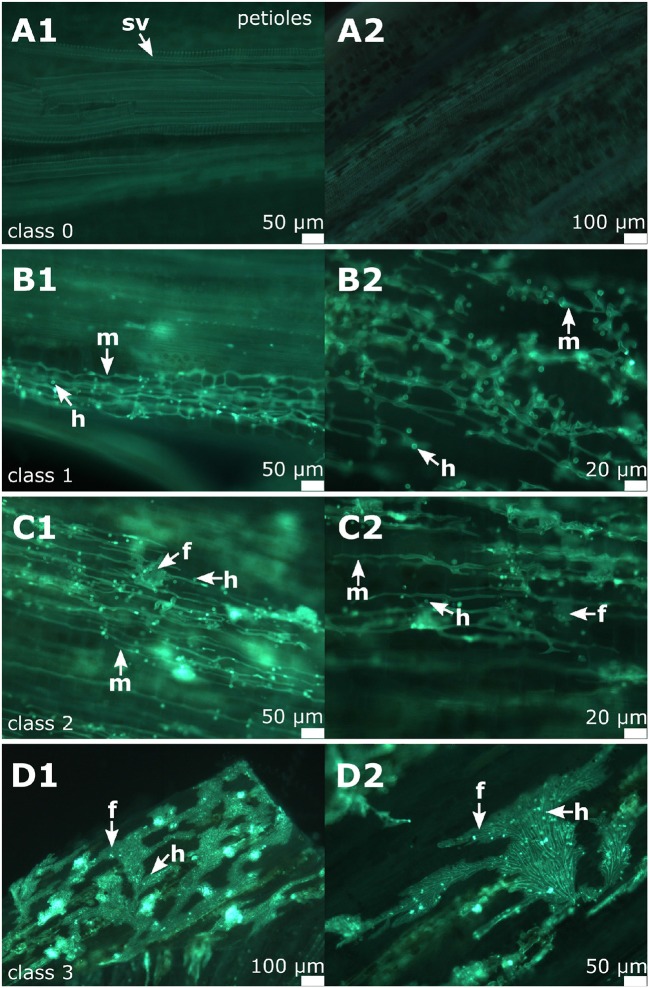
Classification of the degree of infection in petioles. All these example images are from “Pinot noir.” **(A1,A2)** Microscopic image of non-infected petioles (class 0) showing the vascular tissue and surrounding cortical cells. **(B1,B2)** shows microscopic images of infected petioles assigned to class 1. **(C1,C2)** An example of class 2 associated with the formation of fan-shaped hyphae. **(D1,D2)** Samples assigned to class 3 as characterized by the presence of ubiquitous fan-shaped hyphae distributed all over the petioles. m, mycelium; f, fan-shaped hyphae; h, haustoria; sv, spiral vessel.

To provide a reference for further work on *P. viticola* in grapevine, the newly described symptoms of petiole and shoot infections were assigned to different classes. A total of 104 petioles detached from infected leaves of the susceptible cultivars (and 30 petioles from the resistant cultivar) were microscopically evaluated. Half of the samples were frozen for DNA extraction. The other half was cut longitudinally and checked by microscopy. The samples were taken during the entire investigation period from the field plants (May to August 2018) of the susceptible cultivars as well as from the resistant cultivar “Solaris.” Based on the density of infestation in the susceptible cultivars, infections were divided into four classes (Figures 6A–D and Table 1). The degree of infestation was classified as follows. The first class (class 0) (Figures 6A1,A2) shows non-infected samples without any pathogen infection (17 samples). The second class (class 1) relates to an infection with growing mycelium and regular haustoria formation (Figures 6B1,B2) (60 of the 104 evaluated samples). Since the appearance of the fan-shaped hyphae indicates some progressing infestation, the next group (class 2) (Figures 6C1,C2) is characterized by the presence of fan-shaped hyphae (21 samples). The last class (class 3) (Figures 6D1,D2) was assigned to all the heavy infections that formed ubiquitously fan-shaped hyphae. This class comprised six samples. The four different susceptible grapevine cultivars studied did not exhibit any difference concerning the distribution of these infection classes. The samples of “Solaris” presented no signs of infection with *P. viticola*.

**Table 1:** Overview of the classification of infected petioles and shoot tips.

**Degree of infection (class)**	**Petioles**	**Shoot tips**
0	17	1
1	60	33
2	21	16
3	6	1

This table lists the total values from investigated petioles (104) and shoot tips (51) of susceptible cultivars. The valuation unit 0 (class 0) represent all non-infected samples, class 1 covers the samples that show an infection with mycelium growth, haustoria formation but no the fan-shaped hyphae. Class 2 and 3 include all infected materials that show different levels of the characteristic fan-shaped hyphae (cf. Figure 6).

**Figure 7:**
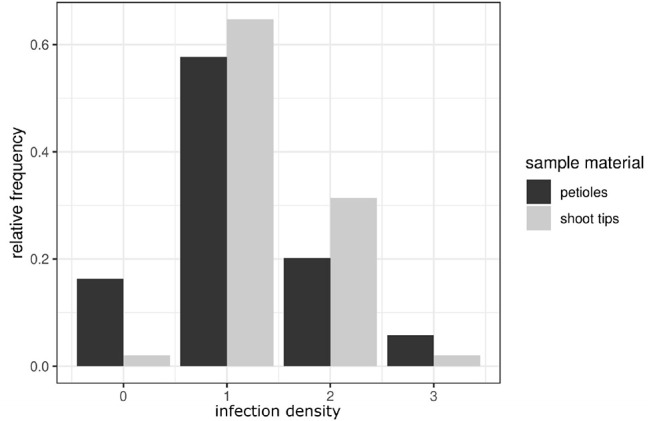
Comparison of the infection classes observed in petioles and shoot tips. The relative frequency of infection classes in shoot tips (presented in light gray) compared to the classification frequencies in petioles (dark gray bars). The bars represent the distribution of the density of infestation in the petioles and shoot tips in susceptible cultivars. Classes range from class 0 (no infection) to class 3 (high density of a *P. viticola* infection).

For the classification of shoot tip infections, shoots bearing at least one of the branching leaves with symptoms of *P. viticola* infection were studied. The pathogen was detected in 50 of the 51 shoots examined. The classification followed the one suggested for petioles (four classes, Figure 7). Again, the individual classes differed concerning the intensity of infection as deduced from the presence of various pathogen-specific elements. The largest groups were classes 1 and 2 (Table 1). The first class (class 0) contains all non-infected samples, the second class (class 1) includes all samples that showed a regular infection with *P. viticola*, where moderate mycelium growth and haustoria formation were detectable. The last two classes (class 2 and 3) differed in the frequency and strength of characteristics of the fan-shaped hyphae (Figure 6). In general, the hyphae seemed to extend along vascular tissue bundles. “Solaris” showed no indication of *P. viticola* infection in all the investigated (*n* = 12) shoot tips.

### 3.5. The Berries and the Seeds

**Figure 8:**
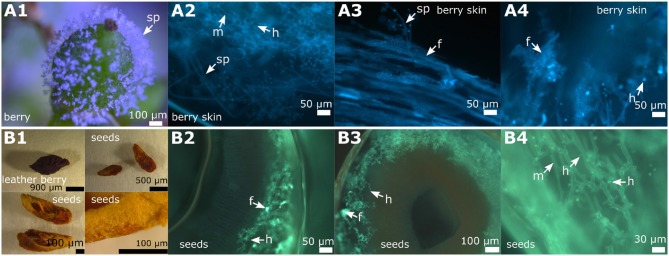
Overview of DM infected berries and seeds in grapevines. Examples of microscopic images from the different stages of a berry infection (cultivar “Riesling”). **(A1–A4)** The young berries with emerging sporangiophores **(A1)** showed the formation of fan-shaped hyphae in the microscopic image **(A2–A4)**. The formation of haustoria by the regular, but also by the fan-shaped hyphae, is clearly recognizable. **(B1–B4)** The first picture **(B1)** shows a berry that has been severely damaged by the pathogen and seeds derived from it. The seeds were separated and partly cut longitudinally and partly cut transversally. The microscopic images **(B2–B4)** represent cross sections of the seeds. Infiltration by the pathogen by typical mycelium (shown in green) is evident with all the typical pathogen elements. The pathogen spread along the inner side of the exotesta. The last microscopic picture **(B4)** shows a longitudinal section through the seed. m, mycelium; f, fan-shaped hyphae; sp, sporangiophores; h, haustoria.

Infected berries were studied at two developmental stages during the season (June 11, 2018 and August 27, 2018). The first examination occurred at the early stage of ripening, when the individual berries are still firm and the stomata apparently still open (BBCH 71, Lorenz et al., 1994) or transformed to open lenticels that permit the emergence of sporangiophores. These berries were collected in the beginning of June. Macroscopically, the infection caused by *P. viticola* showed the typical growth with sporangiophores surrounding the whole berry (Figure 8A1) on susceptible cultivars. The growth of the mycelium within the berry and the formation of pathogenic structures was investigated by layering transversal sections (thickness 2 mm) through the stained samples (Figures 8A2–A4). Mycelium growth proceeded through the complete inside berry with the formation of numerous haustoria. As observed in the leaves, the formation of fan-shaped hyphae was evident in the berries. However, in contrast to leaves, the fan-shaped hyphae in the berries formed haustoria (Figure 8A4). This special form of hyphae was found in all the berries examined (*n* = 100) from susceptible grapevines. The resistant cultivar “Solaris” did not exhibit any symptoms at the berries at this time [as confirmed by microscopy of ten berries of “Solaris” (Figure S1)].

The second date of berry investigation was at the end of August 2018 when the berries were close to ripeness (BBCH 81, Lorenz et al., 1994). Berries of the susceptible cultivars (*n* = 100) showed the typical late symptoms of DM infection. Grapevine berries develop ontogenetic resistance during ripening due to a conversion of their stomata into lenticels and clogging of the stomatal openings (Jackson, 2008; Gindro et al., 2012). DM symptoms observed late in the season therefore result from earlier berry infection, probably taking place already at or even before the flowering stage (Gindro et al., 2012). In the resistance cultivar “Solaris” no “leather berries” could be detected.

**Figure 9:**
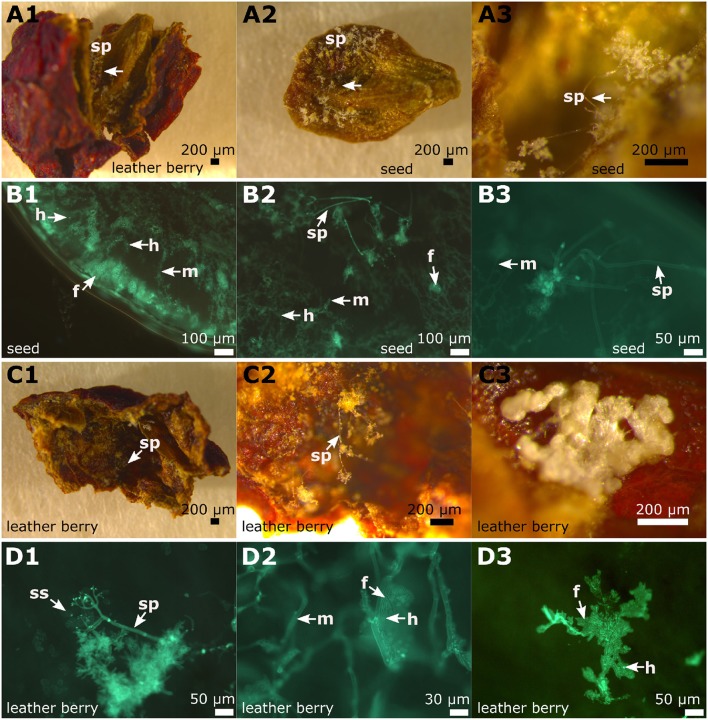
Microscopy of infected berries and seeds. The inspection of the late infected berries (example from “Pinot blanc”). **(A1–A3)** Binoscopic image **(A1)** of an infected berry and its seeds with typical signs of late *P. viticola* infection. Inside the berry, a seed is completely covered with sporangiophores. These grow mainly at the concave side of the seed. **(B1–B3)** Microscopy of transversal section of a seed with its surface covered with sporangiophores. The seed is completely infiltrated by hyphae, that produce haustoria and fan-shaped hyphae. The surface shows typical sporangiophores and hyphae inside. **(C1–C3)** A frequently appearing picture: The inner skins (85% of investigated infected berry skins) of the berries are covered with sporangiophores and at irregular intervals “cushion-like” hyphae appear. **(D1–D3)** According to microscopic evaluation the “cushion-like” hyphae correspond to fan-shaped hyphae. m, mycelium; f, fan-shaped hyphae; sp, sporangiophores; h, haustoria; ss, sporangia.

The “leather berries” were shrunken in volume, dehydrated and had turned brown (Figures 8B1, 9A1). No sporangia were detectable macroscopically. However, in microscopy, berry sections stained with aniline-blue after KOH treatment exhibited the same picture as the younger berries collected at the earlier date. In the whole inside berry area *P. viticola*-specific mycelium was found. Haustoria and fan-shaped hyphae were identified. These pathogen-specific elements were present in all the infected berries investigated. At the inside of the berries, sporangiophores developed (Figures 9A2,A3,C1,C2). In addition, within the infected berries, whitish to yellowish clearly defined structures (“cushion”-like lobed hyphae) were found (Figures 9C3,D3). These show the structure and shape of the fan-shaped hyphae, but appear to be covered by some glossy and firm substance (Figure 9C3). They are only observed along the hypodermis (the inner tissue layer of the berry skin (Jackson, 2008). Further microscopy indicated that they are associated with the fan-shaped hyphae (Figure 9D3). In an examination of macroscopically uninfected berries (*n* = 10) for comparison, no such structures were found indicating that they are specific for the pathogen.

### 3.6. A Close Look at the Seeds

At the same two developmental stages when the berries were studied, the seeds of these berries were examined for the presence of pathogen-specific structures. At the first stage of the infected young berries, no evidence of *P. viticola* infection was found in any of the seeds (*n* = 50) examined.

In contrast, the 50 seeds of the “leather berries” collected in August showed clear indications of *P. viticola* infection (Figures 8B2–B4, 9D1,D2). Transversal sections were made to assess the depth of infestation and longitudinal sections were performed to better recognize the pathogen-specific elements. *P. viticola* infection structures could be detected in all the seeds investigated. The transversal sections showed different penetration depths of the mycelium. The pathogen penetrated around the first 40% of the seed volumina (counted from the exotesta to the inner lumen) in the majority of the transversal sections. A small number of the seeds was completely penetrated by mycelium (Figures 9B1–B3). The smallest fraction of the seeds showed colonization with a penetration depth of only around 10%.

In the longitudinal seed sections, depending on the position of the section, the mycelium was present with all its typical structures. The course of the mycelium between the cells was well-observable. Some of the haustoria were depicted individually in differentiated cells, while sometimes the mycelium that formed them could not be stained at the same focus level in a section. In general, the course of the hyphae in all samples was strongly directional without branching. In some cases, the hyphal structure changed to the fan-shaped hyphae and later reverted to the typical tubular hyphal structure again. These altered hyphal structures did not show up in all investigated seed samples. They seemed to occur exclusively during heavy pathogen infestation. The examination of seeds infected with *P. viticola* did not reveal any endosperm or embryo. Infected seeds therefore are biologically dysfunctional. However, endosperm was present in non-infected seeds of the same developmental stage. In the seeds of uninfected berries (*n* = 10) checked for control, no pathogen-specific structures could be found.

## 4. Discussion

Due to its high relevance in viticulture and grapevine resistance breeding, *Plasmopara viticola* has been subject to numerous studies yielding currently (as of May 14, 2019) 3,452 publications cited in “Web of Science” (Clarivate Analytics). These research papers deal with many different aspects, including fungicide resistance, epidemiology, ultrastructure or grapevine resistance responses. Due to the recent elucidation of the *P. viticola* genome sequence, studies on genes involved in pathogenesis and secreted effectors became feasible (Yin et al., 2017; Brilli et al., 2018; Dussert et al., 2018). However, most of these studies used infected leaf tissue only.

It has been reported that *P. viticola* leaf infections are restricted to the intercostal fields (Burruano, 2000; Unger et al., 2007). The vascular tissue in leaf veins seems to present a physical barrier that impedes the colonization of adjacent areas of the leaf tissue, resulting in a “mosaic” pattern of leaves arising from several independent infection foci. Since the pathogen infects through stomata and primarily colonizes the spongy mesophyll, this conception may be appropriate in many cases of early infestation (Burruano, 2000). However, in this study a special structure of “fan-shaped” hyphae able to traverse the leaf veins was found already 6 days after experimental inoculation. The appearance of these structures was confirmed in natural infestations in the field on grapevine cultivars of international significance and high relevance in Germany. In the leaves, these special hyphae do not form haustoria. After crossing the veins, they proceed to the next intercostal field and resume their regular tubular shape forming again haustoria to retrieve the nutrients from their host cells. They appear as a morphological structure especially adapted to overcome a mechanical barricade. Vascular tissue in leaf veins is surrounded by a protective “bundle sheath” and thick-walled cells (Jackson, 2008). It contains fortified tissue and dead xylem vessels that obviously prevent the normal colonization by the oomycete through appressoria and haustoria.

Such fan-shaped hyphae traversing the leaf veins have been described earlier by Kortekamp (2005) and are marginally mentioned in the work of Trouvelot et al. (2008), but did not deserve much attention due to other properties of *P. viticola* that were in the focus of these former studies. Interestingly, a histological study on *Peronospora rubi*, an oomycete pathogen of *Rubus* sp., exhibits very similar structures of fan-shaped or “fasciated” hyphae (Williamson et al., 1995). In their description, the authors assume that these fasciated hyphae may be built by anastomosis of several hyphae that come together closely in the infected tissue. From the microscopy presented here, it rather seems that the fan-shaped hyphae emerge by widening and formation of lobes of individual or few tubular hyphae. The fan-shaped hyphae exhibit some longitudinal stripes of unknown nature. The presence of microtubules and microfilaments forming longitudinal arrangements along the hyphae has been described in oomycetes (Hardham, 2007). It may be speculated that the longitudinal stripes observed in the fan-shaped hyphae of *P. viticola* are related to such cytoskeletal elements. However, specific histological staining is necessary in the future to verify this assumption. As demonstrated here, the mycelium of *P. viticola* spreads to petioles and shoot tips. In these tissues, it appears to grow in the space between cortical cells, growing in longitudinal direction in parallel to vascular bundles. In agreement with very early observations (Gregory, 1915), the oomycete does not seem to attack xylem or phloem tissue.

In this microscopic study, the growth of the oomycete seems to proceed through the cambium (Figure 4) in young shoots. The aniline blue stain can be applied to indicate the presence of callose. This β-1,3-glucan is present in the vascular system and frequently synthesized at original fungal infection sites as defense reaction to shut-off the invading pathogen (e.g., Trouvelot et al., 2008). However, the pretreatment by KOH during the preparation of the microscopic samples increases the affinity of the pathogenic elements to aniline blue. It therefore renders the staining method rather specific for fungi and oomycetes (Hood and Shew, 1996). The possibility that the fluorescence observed in the cambium is due to callose deposits instead of oomycete structures is therefore highly unlikely. Furthermore, in *Arabidopsis* it was reported that callose is specifically synthesized in the phloem tissue of the vascular elements (Xie et al., 2011). In the experimental set employed in this study, there was no epifluorescence signal from phloem or from the tissue surrounding the stomata that serve as original infection sites for *P. viticola*.

According to recent genome sequence analysis, the oomycete has several genes encoding secreted pectin esterases and pectate lyases and other cell wall degrading enzymes (Yin et al., 2017; Dussert et al., 2018). These may function to loosen the pectinaceous cell joints in middle lamellae and permit intrusion into the host tissue.

Mycelium in petioles again forms fan-shaped hyphae. These are pronounced in heavy infections, where they may enable the pathogen to cross the physical barrier constituted by the vascular tissue (as in the leaf veins), colonizing the whole petiole in this way. Similar observations apply to the shoot tips, which are also colonized by intercellular growth in longitudinal direction along the conducting tissue. In both cases, plenty of haustoria are formed that allow the pathogen to retrieve its nutrients from neighboring parasitized cells. In both organs, petioles and shoot tips, infection density varied throughout the complete epidemic and was independent of the epidemic progress from the first starting infections to the late infections in the year. The pathogen colonizes its host to gain nutrients. It seems to spread via the leaf veins and leaf stems to the shoot tips. In contrast to the fan-shaped hyphae in leaf tissue, these special hyphae appearing in petioles and shoot tips develop haustoria. Development of sporangiophores was not observed in all of the investigated samples in either petioles or shoot tips.

The fan-shaped hyphae are also evident in young berries, leather berries and their seeds. It seems that they develop, whenever the pathogen tries to overcome a physical barrier (like vascular tissue or other strong tissue layers in the host plant). From the microscopic pictures presented here, one might get the impression that these structures arise to generate some pressure to surmount a physical obstacle hindering proliferation of the pathogen. If this hypothesis would be true, these fan-shaped hyphae may be functionally reminiscent of the well-known emergence of appressoria from infecting hyphae, that attach to host cells in a slightly lobed configuration to invade the plant cell by the formation of haustoria.

The fan-shaped hyphae extended along the inner berry skins of “leather berries.” In this tissue, they appeared to be covered by some glossy and firm, whitish to yellowish substance. The nature of this layer is unknown. However, it has been noted that *P. viticola* has active genes for the synthesis of chitin which seem to play a role during pathogen proliferation (Werner et al., 2002). In the oomycete fish pathogen *Saprolegnia* chitin synthesis at growing hyphal tips has also been demonstrated (Guerriero et al., 2010). Therefore, there is a possibility, that the external layer observed is constituted of chitin. However, *P. viticola* has also been found to excrete quite a number of other substances (Yin et al., 2017; Dussert et al., 2018) and to initiate some specific gene expression during infection (Luis et al., 2013). This opens a wide range of possibilities to explain this glossy surface appearance possibly caused by some secreted compound. In the oomycete *Phytophthora infestans* (causing late blight in potato) it was found that synthesis of cellulose is prerequisite for appressorium formation (Grenville-Briggs et al., 2008). In *Peronospora parasitica*, the causal agent of DM in various *Brassica* spp., a fibrillar extracellular matrix of carbohydrates has been reported for appressoria (Carzaniga et al., 2001). Most interestingly, again in *P. infestans*, formation of mucin-like proteins during the biotrophic phase and appressorium formation was found (Görnhardt et al., 2000). These proteins are extremely hydrophilic, viscoelastic and sticky. In humans, they protect epithelial cells from desiccation and abiotic or biotic damages. Thus, resuming the hypothesis, that the fan-shaped hyphae are structurally similar to appressoria, there are several possibilities that could explain their glossy appearance by some secreted component or a mixture of several of them. However, elucidation of the chemical nature of this apposition awaits further analysis.

*Plasmopara viticola* proliferating on the inside of leather berry skins develops sporangiophores. They can release sporangia since the berry skin is broken and open to the environment. Sporangiophores were also identified on the outside of the infected seed mummies. Seeds from that stage of infection did not contain any endosperm or embryo (Figure 8B1). Therefore, they are not functional and seed transmission of the disease is highly unlikely.

From the observations here, it seems that *P. viticola* is able to infect its host in a systemic way. It can spread from original infections on leaf stomata or very young fruits to petioles and shoots, respectively to developing berries. This type of infestation is also observed in *Botrytis cinerea* (teleomorph *Botryotinia fuckeliana*), the causal agent of gray mold (McNicol et al., 1985; Bristow et al., 1986; Williamson et al., 2007). An explicit criterion to distinguish the two pathogens on microscopic level is the formation of haustoria as clearly shown here for *P. viticola* (Figures 1C,D). The fan-shaped hyphae described here also formed these hausoria. In contrast, *B. cinerea* never produces haustoria. Therefore, any possible confusion with *B. cinerea* infection can be excluded. In addition, the amplified ITS region sequence confirms the identity of *P. viticola* in the leaves.

To further exclude any confusion between the two pathogens in the fruits, berries infected with *B. cinerea* were studied for comparison. The color of the sporangiophores and the branching of the upper part of the sporangiophores were evaluated as delimiting characteristics. The color of the sporangiophores in *P. viticola* is translucent colorless, while they are black in *B. cinerea* (Figure S3). The branching of the sporangiophores is finely defined in *P. viticola* and the individual sporangia can be clearly distinguished from each other, while they stick more densely to each other in *B. cinerea* and appear gray in color. *P. viticola* shows small, less branched sporangiophores in which the sporangia appear translucent. In addition, the sporangiophores of *P. viticola* are separated from the mycelium by septa, but the mycelium is coenocytic. In contrast, *Botrytis* hyphae show regular septation. In the microscopic picture, *P. viticola* in the berries shows growth through the entire berry skin and the flesh producing many haustoria. In contrast, *B. cinerea* does not attack seeds and endosperm and untouched embryos were present in the seeds of *Botrytis*-infected berries. This comparison excludes any doubts about the identity of the *P. viticola* pathogen investigated here.

In conclusion, this study shows that *P. viticola* is able to spread throughout the whole grapevine plant. It forms special fan-shaped, lobed hyphae to overcome physical obstacles like conducting tissue. The frequent appearance of haustoria suggests that this pathogen absorbs plenty of nutrients throughout its colonization of the host plant. This may enable it to survive even in the case of poor host plant growth conditions. This finding raises the question if over-wintering of *P. viticola* may occur in the mycelial state in wood or dormant buds. Such mycelium could play a role for initial infection in the following year. This question should deserve further attention and research.

## Data Availability

All datasets generated for this study are included in the manuscript and/or the Supplementary Files.

## Author Contributions

SF conducted the experiments and performed the microscopic analyses. EZ directed the study and discussed results. SF and EZ wrote the manuscript.

### Conflict of Interest Statement

The authors declare that the research was conducted in the absence of any commercial or financial relationships that could be construed as a potential conflict of interest.
