# Supplementary material for: Colonization of Different Grapevine Tissues by Plasmopara viticola—A Histological Study
Source: Front Plant Sci. 2019 Jul 24;10:951. doi: 10.3389/fpls.2019.00951 (PMC6667660; doi:10.3389/fpls.2019.00951)
Supplement: Supplementary file 1 [file Data_Sheet_1.PDF]

Supplementary Material: Colonization of grapevine by *Plasmopara viticola* – a histological study

1 SUPPLEMENTARY TABLES AND FIGURES

1.1 Figures

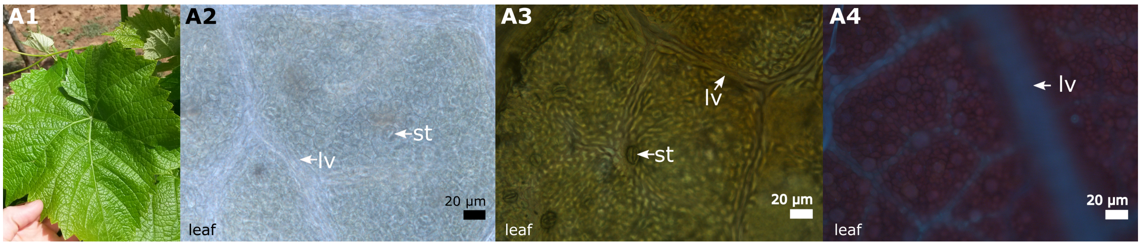

Figure S1. Non infected leaf material of the resistant cultivar 'Solaris'
The samples of 'Solaris' were collected in a non-sprayed experimental plantation. **A1)** The image shows the cultivar 'Solaris' with no signs of any infection of *P.viticola* on the leaf. **A2-4)** The microscopic evaluation shows the absence of pathogenic elements as well. **A2)** represents a brightfield-, **A3)** a phase-contrast- and **A4)** a fluorescence (KOH-treated aniline blue stained) microscopic image of the leaf. Abbreviations: if, intercostal field; lv, leaf vein; st, stomata

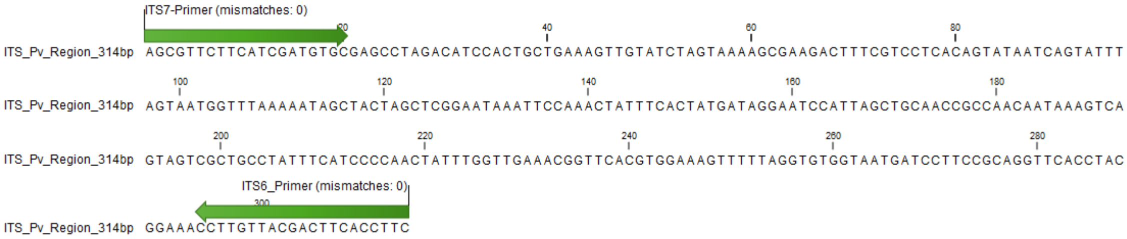

Figure S2. ITS-region of *P. viticola*
ITS-region and the ITS primers 6 and 7 (Cooke et al., 2000)

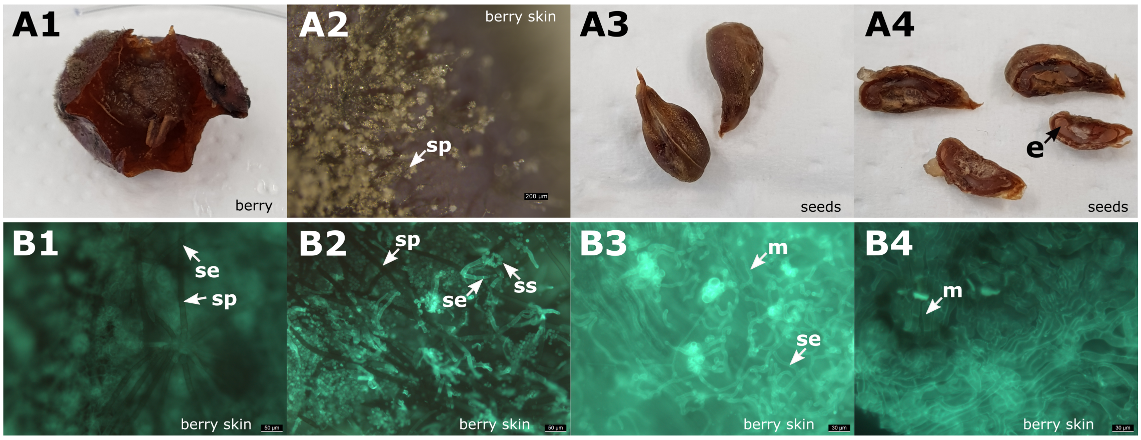

Figure S3. Differentiate a *P. viticola* infection from *B. cinerea* one
A1-4) Overview of a *Botrytis* infected berry and the seeds inside of the cultivar 'Müller-Thurgau'. The berry skin is covered with sporangiophores all over **(A1,2)**. They are dark brown to black in coloration. The seeds (**A3**) do not show any sign of infection. When they are open for inspection, their endosperm and embryos appear undamaged (**A4**). **B1-4)** This microscopic view of the berry skin surface shows a huge amount of dark brown sporangiophores **(B1, 2)** with frequent septa. This septation appears also in the mycelium. In contrast to *P. viticola* infection, there are no haustoria formed. Abbreviations: sp, sporangiophores; m, mycelium; e, endosperm; se, septum; s, sporangiospores

REFERENCES

Cooke, D., Drenth, A., Duncan, J., Wagels, G., and Brasier, C. (2000). A molecular phylogeny of *Phytophthora* and related oomycetes. *Fungal genetics and biology* 30, 17–32. doi:10.1006/fgbi.2000.1202
